# Risk factors associated with ST-segment elevation myocardial infarctions among young patients treated in the Mid-West of Ireland: a case series report using secondary data

**DOI:** 10.1007/s11845-025-03965-w

**Published:** 2025-05-29

**Authors:** Sandra N. Nantumbwe, Roisin Gardiner, Thomas J. Kiernan, Niamh M. Cummins

**Affiliations:** 1 Faculty of Education and Health Sciences, School of Medicine, University of Limerick, Limerick, Ireland; 2 Health Research Institute, University of Limerick, Limerick, Ireland; 3 Department of Cardiology, University Hospital Limerick, Dooradoyle, Ireland; 4 Department of Paramedicine, Faculty of Medicine, Nursing and Health Sciences, Monash University, Melbourne, VIC Australia

**Keywords:** Cardiovascular disease, Risk factors, Smoking, STEMI, Young adults

## Abstract

**Background:**

ST-segment elevation myocardial infarction (STEMI) occurs when the myocardium has been damaged due to an acute occlusion of a coronary artery. STEMI commonly presents in older populations; however, little is known about the prevalence and risk factors among young patients. This is the first study on STEMI in young patients in Ireland.

**Aims:**

To determine the prevalence of STEMI and explore the associated risk factors in a cohort of young adults aged 45 years and below in the Mid-West of Ireland.

**Methods:**

This is a case series report using secondary data analysis of data collected from patients aged 45 years or below, hospitalized at University Hospital Limerick between January 2012 and December 2019. All patients who had a primary diagnosis of STEMI during this time period were eligible for inclusion. Clinical data were collected via electronic records and included demographic, clinical, and biomarker variables.

**Results:**

The sociodemographic characteristics of the STEMI patients revealed that a majority were male (90%), overweight or obese (63%), smokers (69%) and lived in urban areas (74%). Over half of the patients (53%) were diagnosed with hypertension, 69% had a family history of cardiovascular disease (CVD) and 40% had diabetes diagnosis. After initial hospitalization, 64% were readmitted for another procedure.

**Conclusions:**

This study identified cardiovascular risk factors among STEMI patients in Ireland as male, smoking, family history of cardiovascular disease, and being overweight or obese. This study hopes to increase awareness of young STEMI patients and inform health promotion initiatives within this cohort.

**Supplementary Information:**

The online version contains supplementary material available at 10.1007/s11845-025-03965-w.

## Introduction

Cardiovascular disease (CVD) is the leading cause of mortality globally; accounting for an estimated 17.9 million deaths every year [1]. This represents 32% of all deaths and a third of these are premature, occurring in patients below 70 years of age [1]. CVD is a collection of various blood and heart diseases that include coronary heart disease (CHD), rheumatic heart disease, cerebrovascular disease, peripheral arterial disease, among others [1]. In 2015, more than 85 million people in Europe were living with CVD [2] while 7.4 million deaths were caused by CHD alone [3]. In the same year, the European Union (EU) spent over 210 billion euros on CVD with over 50% (111 billion euros) spent on healthcare and 26% in losses of productivity [4]. By 2030, CVD mortality is expected to reach 23.6 million and remain the leading cause of mortality over the next few decades. In Ireland alone, CVD kills about 9000 people yearly yet 80% of the premature CVDs are preventable [5].

Myocardial Infraction (MI) is one of the most common types of CHD and occurs from the full or partial occlusion of a coronary artery, significantly impairing blood flow to the myocardium in the territory supplied by that artery, causing it to become infracted [3]. In 2020, approximately 20% of individuals in Ireland that died of diseases of the circulatory system were due to an MI [6]. In the Mid-West region alone, 215 patients died from an MI in 2019 [7]. International evidence has shown that men carry the highest prevalence of MI [3] and disability adjusted life years (DALYs) are twice as high in men than women [4]. The mean age for MI is 65 and 72 for males and females respectively [8]. Almost three-quarters (72.5%) of deaths due to diseases of the circulatory system occurred in patients aged 75 and over [7]. A ST-segment elevation myocardial infarction (STEMI) is a type of MI where the coronary artery occlusion leads to transmural myocardial ischaemia and as a result, myocardial necrosis.

STEMI accounts for approximately 25% of all MIs [9] and has a mortality rate of 4–24% [10]. More than 3 million individuals develop STEMI each year and more than 4 million people represent STEMI pathology [11]. Some studies of risk factors of STEMI in the general population suggest male gender, hypertension, smoking, diabetes, and family history as risk factors; however, it is unknown whether these are the same for the younger adults (aged below 65 years) [12]. While it was formerly known as a disease of the elderly, there is an epidemiological transition with an increasing prevalence of STEMI in young patients [13]. Globally, little is known about the prevalence of STEMI in younger patients and the associated risk factors. Some studies have suggested younger patients largely have no ischemic preconditioning and therefore STEMI can progress more quickly than in older patients [13]. Existing STEMI studies often group young and older patients together [12]. This research study is innovative in that it solely focuses on exploring the characteristics of young patients who have suffered a STEMI. By 2025, through a global action plan, WHO member states aim to reduce the number of NCD premature deaths by 25%, with two target areas focusing precisely on the prevention and control of CVDs [1]. As this is the first study on STEMI in young patients in Ireland, it is hoped that the findings will shed more light on this novel area and reduce premature deaths in the long term.

Therefore, this study aims to identify the prevalence of CVD risk factors in young patients (≤ 45 years) presenting with STEMI at University Hospital Limerick (UHL) between 2012 and 2019. It specifically seeks to explore the associated cardiovascular risk factors in this cohort.

## Methodology

### Ethics statement

The collection of this data for research was approved by the ethics committee of the University of Limerick (UL) Hospitals Group Research Ethics Committee (REC) at UHL. The data was collected by the UHL cardiac rehab nurses between 2012 and 2019 and all patients provided informed consent. The study complies with the ethical guidelines of the Declaration of Helsinki. A STROBE statement was included to enhance completeness and transparency of this case-series study.

### Study participants

A retrospective, single centre, case series review of the case notes of 146 young STEMI calls to UHL over an 8-year period between January 2012 and December 2019. The diagnosis of STEMI was made by the treating consultant cardiologist at UHL based on clinical history, electrocardiography findings, laboratory results and angiographic results in line with European Society of Cardiology (ESC) Guidelines. The data was originally collected to analyse the outcomes of Percutaneous Coronary Intervention (PCI) being performed at the hospital.

### Inclusion and exclusion criteria

The inclusion criterion were patients who had a primary diagnosis of a STEMI and were aged 45 years or below. The majority of studies use 45 years as the cut-off age to define young patients with acute MI or CAD [13]. There were no specific exclusion criteria for this study.

### Study variables

A retrospective review of patient notes and the UHL laboratory system was performed to determine risk factors. The demographic variables analysed in this study include sex, age, BMI, smoking status, place of residence, socioeconomic status (SES) and residence distance from hospital. Smoking was defined as being a current smoker or having a significant smoking history. Patients were classified unto urban and rural based on the definition provided by the Central Statistics Office of Ireland. SES of the patients was determined using the 2017 Pobal Trutz Haase Deprivation Index. The index is a validated, census-based tool using the latest 2016 national census [14].

The clinical variables include hypertension, diagnosis of diabetes as derived from the levels of glycated haemoglobin (HbA1c), family histories of CVD, premature coronary artery disease (CAD), and hypercholesterolaemia, readmissions to hospital and post-discharge survival. Hypertension was defined as a systolic pressure ≥ 140 mmHg and/or a diastolic pressure ≥ 90 mmHg. HBA1c levels for diagnosis of diabetes are as follows: < 5.7% = normal, 5.7%–6.4% = prediabetes and 6.5% or above = diabetes [15]. Readmission to hospital refers to if the patient was readmitted one or more times to UHL following the PCI procedure. Post-discharge survival means if the patient is dead or alive after being discharged from the hospital. The biochemical variables measured include apolipoprotein (apoA), glycated hemoglobin (HBA1c), Low Density Lipoproteins (LDL), High Density Lipoproteins (HDL), total cholesterol (TC) and thyroglobulin (TG). HDL levels of less than 40 mg/dL, indicating an increased risk of heart disease. The patients completed a questionnaire to obtain information on smoking status and family history.

Due to missing data, the number of patients included in the analysis differed across variables. To ensure transparency, the number of patients corresponding to each variable is presented in all tables.

### Data analysis

Data was exported from Microsoft Excel into IBM SPSS Statistics software for Windows, version 28.0.1.1, where all analysis took place. All variables were initially tested for normality with the Shapiro–Wilk test. Continuous variables were expressed either as mean and standard deviation (SD) or median and interquartile range (IQR), whereas categorical variables were expressed as numbers and percentages. The chi-squared test was used for the categorical data while split files were used to analyze the variables across different categories. Pearsons and Spearman’s correlation tests were used to investigate correlations depending on the distribution. For bivariate comparisons, *t*-tests and Mann–Whitney were utilized for parametric and non-parametric analyses, respectively, while for multivariate comparisons, ANOVAS or Kruskall-Wallis tests were used, depending on the distribution of the data. A *p* value of less than 0.05 was considered significant for all analysis tests [16].

## Results

### Prevalence

Of the 146 young STEMI (aged ≤ 45 years) calls in the database, a total of 101 patients were diagnosed as being true STEMI cases.

### Sociodemographic risk factors

The majority of the patients were male (90%) and 64% of the patients were aged between 41 and 45 (median 42 years: IQR 5). In relation to BMI, almost two thirds of patients (64%) were considered to be overweight (40%) and obese (24%). In relation to smoking status, 69% were smokers, and the majority of smokers (88%) were male. Most of the patients (74%) live in urban areas and 51% live within 24 km of the hospital. With regard to SES, this was recorded as below average (41%), disadvantaged (15%) or very disadvantaged (5%) for 61% of the study population (Table 1).

**Table 1:** Sociodemographic characteristics of STEMI patients aged < 45 years

Characteristic	Frequency, *n*	Percentage, %
Sex (*n* = 78)		
Female	8	10%
Male	70	90%
Age (y) (*n* = 78)		
31–35	4	5%
36–40	24	31%
41–45	50	64%
BMI (kg/m^2^) (*n* = 101)		
Healthy	37	36%
Overweight	40	40%
Obese	24	24%
Smoking status (*n* = 72)		
Smoker	50	69%
Non-smoker	22	31%
Residence (*n* = 39)		
Urban	29	74%
Rural	10	26%
Socioeconomic status (*n* = 39)		
Affluent	4	10%
Above average	10	26%
Below average	16	41%
Disadvantaged	6	15%
Very disadvantaged	2	5%
Distance from hospital (km) (*n* = 39)		
0–24	20	51%
25–49	12	31%
50–74	5	13%
75–99	1	3%
100–124	1	3%

### Clinical risk factors

Almost half of the STEMI patients (47%) were diagnosed with hypertension (56% males and 25% females). Two thirds of the patients (69%) had a family history of CVD and an additional 39% had a family history of CAD and 37% hypercholesterolaemia. After the initial hospitalization, 64% were readmitted to hospital and underwent another procedure (Table 2).

**Table 2:** Clinical characteristics of STEMI patients aged < 45 years

Characteristic	Frequency, *n*	Percentage, %
Hypertension (*n* = 66)		
Hypertension	32	47%
No hypertension	34	53%
Diagnosis of diabetes (HBA1c) (*n* = 71)		
Normal	15	21%
Pre-diabetes	28	39%
Diabetes	28	39%
Family history of CVD (*n* = 62)		
Yes	43	69%
No	19	31%
Family history of CAD (*n* = 62)		
Yes	24	39%
No	38	61%
Family history of hypercholesterolaemia (*n* = 60)		
Yes	22	37%
No	38	63%
ApoA (mg/dl) (*n* = 78)		
< 110	56	72%
110–180	14	18%
> 180	8	10%
Readmission/re-procedure (*n* = 78)		
Readmitted	50	64%
No readmission	28	36%
Post-discharge survival (*n* = 78)		
Alive	41	53%
Unknown	37	47%

The majority (78%) of the young STEMI patients were diabetic or pre-diabetic. A diabetes diagnosis, as indicated by a glycated hemoglobin (HbA1c) above 6.5% was recorded in 39% of the patients, while another 39% had a HbA1c level indicating pre-diabetes diagnosis. Most patients (72%) had an Apolipoprotein A (apoA) level less than 110 mg/dL while 10% were above 180 mg/dL. HDL levels of less than 40 mg/dL, indicating an increased risk of heart disease, were recorded in 78% of patients and only 3% had the recommended levels below 60 mg/dL. Almost a third of patients (28%) had LDL levels ranging between borderline high and very high (Table 3).

**Table 3:** Biomarker characteristics of STEMI patients aged < 45 years (*n* = 78)

Characteristic	Mean	Standard deviation
Total cholesterol (mmol/L)	3.8	2.2
LDL (mg/dL)	94.7	58.3
HDL (mg/dL)	28.8	17.5
	Median	Interquartile range (IQR)
HBA1c (mmol/L)	6.2	1.3
Thyroglobulin (mmol/L)	1.3	1.5
Creatinine (umol/L)	74.5	21

### Subset analysis

**Fig. 1:**
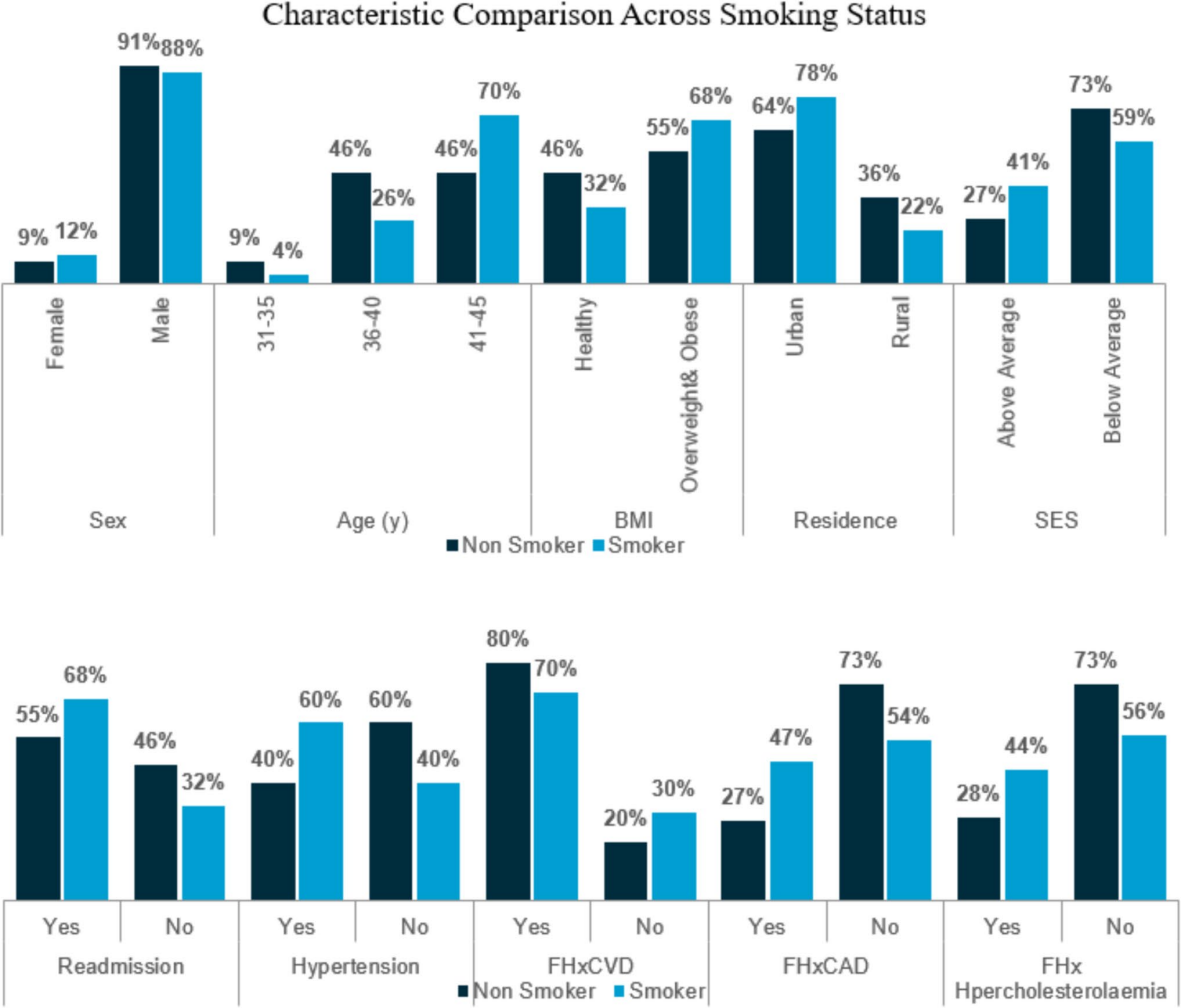
Characteristic comparison across smoking status

Subset analysis was conducted and when comparing across smoking status there was no significant differences in sociodemographic, clinical or biochemical variables (Table 4). The data does illustrate that among smokers, 60% had hypertension, 81% had HDL less than 40 mg/dL, 68% had an apoA less than 110 mg/dL, 68% were readmitted and 70% had a family history of CVD (Fig. 1).

**Table 4:** Characteristic comparison across smoking status

		Non-smoker		Smoker		*p*-value
Characteristic	Detail	*n*	%	*n*	%	
Sex (*n* = 78)	Female	2	9%	6	12%	0.717
	Male	20	91%	44	88%	
Age (y) (*n* = 78)	31–35	2	9%	2	4%	0.136
	36–40	10	46%	13	26%	
	41–45	10	46%	35	70%	
BMI (*n* = 101)	Healthy	10	46%	16	32%	0.274
	Overweight or obese	7	55%	34	68%	
Residence (*n* = 39)	Urban	7	64%	21	78%	0.369
	Rural	4	36%	6	22%	
SES (*n* = 39)	Above average	3	27%	11	41%	0.269
	Below average	8	73%	16	59%	
Readmission (*n* = 78)	Yes	12	55%	34	68%	0.274
	No	10	46%	16	32%	
Hypertension (*n* = 66)	No	12	60%	20	40%	0.129
	Yes	8	40%	30	60%	
FHxCVD (*n* = 62)	Yes	12	80%	30	70%	0.445
	No	3	20%	13	30%	
FHxCAD (*n* = 78)	Yes	4	27%	20	47%	0.179
	No	11	73%	23	54%	
FHx hypercholesterolaemia (*n* = 60)	Yes	4	27%	18	44%	0.242
	No	11	73%	23	56%	
HbA1c (mg/dl) (*n* = 71)	Normal (< 5.7)	8	38%	9	21%	0.267
	Pre-diabetes (5.7–6.4)	5	24%	17	39%	
	Diabetes (≥ 6.5)	8	38%	18	41%	
ApoA (mg/dl) (*n* = 78)	High risk (< 110)	13	82%	34	68%	0.447
	Normal (110–180)	2	9%	10	20%	
	Recommended (> 180)	2	9%	6	6%	
HDL (mg/dl) (*n* = 78)	High risk (< 40)	13	68%	38	81%	0.224
	Normal (40–59)	6	32%	7	15%	
	Recommended (≥ 60)	0	0%	2	4%	
LDL (mg/dl) (*n* = 78)	Optimal (< 100)	5	18%	25	54%	0.221
	Near optimal (100–129)	5	26%	10	22%	
	Borderline high (130–159)	6	32%	8	17%	
	High (160–189)	2	11%	1	2%	
	Very high(≥ 190)	1	5%	2	4%

Among those with a family history of CVD (Supplementary Table 1), 61% were aged between 41 and 45, 65% were overweight or obese, 81% lived in urban areas, 63% had an apoA less than 110 mg/dL and 88% had HDL less than 40 mg/dL. Furthermore, there were significant differences (*p* < 0.05) in HbA1c, HDL, FH CAD and FH hypercholesterolaemia associated with a FH of CVD.

## Discussion

To our knowledge, this is the first Irish research study focused specifically on the characteristics of young patients aged ≤ 45 years suffering STEMI. Previous studies in Ireland have predominantly focused on older patients or did not explore the specific characteristics of young patients with STEMI. The findings of our study indicate that there were 101 true young STEMI patients (≤ 45 years) treated in the Mid-West during the study period from 2012 to 2019. In the Irish Heart Attack Audit (IHAA), a recent national report of 6616 STEMI cases, the median age of patients was 63 years [17]. The IHAA reported that STEMI was the first manifestation of CHD for 75% of patients. This serves to highlight the importance of identifying the risk factors for STEMI in younger patients from a public health perspective. Our findings indicate that the majority of young STEMI patients were male (90%), overweight or obese (63%), smokers (69%) and lived in urban areas (74%) of below average SES (61%). Almost half of the patients (53%) were diagnosed with hypertension, 69% had a family history of CVD and 40% had a diabetes diagnosis. After the initial hospitalization, 64% were readmitted for another procedure. In this study, males were at increased risk of STEMI in these young patients comprising 90% of this cohort. The gender distribution imbalance observed is possibly associated with the protective effects of estrogen in the prevention of atherosclerosis [18].

CVD morbidity and mortality has been shown to be elevated in individuals who are overweight and obese (Luma and Ahmad, 2011). According to the Healthy Ireland 2015 Survey, 60% of the Irish population aged 15 years and over are either overweight or obese — 37% are overweight and a further 23% are obese [19]. Our study found that almost two thirds of patients (63%) were considered overweight (40%) and obese (24%). While those who were overweight in our STEMI cohort was higher than the national cohort, those who were obese were closely higher in the national population. Despite its established undesirable impact on general and cardiovascular health, multiple studies have demonstrated better survival for overweight and obese patients after a STEMI compared to patients with a normal BMI [20]. The mechanisms underlying this “obesity paradox” are controversial and remain unconcluded [21]. Suggestions for this paradox have been proposed, many focused around the neuroendocrine and metabolic effects of adipose tissue leading to a more favorable profile for higher BMI patients against catabolic stress, inflammation, cachexia, and frailty [22].

Smoking is the leading cause of preventable death in Ireland with over 100 people dying from tobacco-related disease every week [23]. According to the Healthy Ireland Survey, 17% of the population are smokers, with 19% of men currently smoking compared to 16% of women. The prevalence of smoking in our STEMI cohort was much higher compared to this national survey with an overall prevalence of 69% smokers of which 88% were males and 12% females. This finding is in agreement with the IHAA report which highlighted that 64% of heart attacks in patients aged below 40 years were active smokers at the time of the heart attack [17]. International studies have also reported that smoking prevalence ranged from 47 to 83% in young patients with MIs [24]. Smoking adversely affects the cardiovascular system by introducing toxins that promote blood hypercoagulability, increasing the risk of thrombosis and vascular blockages [25].

Recently, a retrospective cohort study demonstrated that among young patients with MI, those who stopped smoking after diagnosis of the MI had remarkably lower cardiovascular and all-cause mortality than those who did not quit smoking [24]. Findings on national health policies aimed at raising the legal smoking age revealed that a delay in smoking initiation among youth would, in the long term, reduce the prevalence of smoking in the adult population [24]. Ireland has various measures in place to strengthen the prevention of smoking initiation and promoting cessation as it intends to become tobacco-free by 2025 [23]. This study significantly highlights the prevalence of smoking in young STEMI patients. Smoking is a modifiable risk factor and therefore health promotion policies related to smoking cessation may reduce the incidence of smoking in Ireland and potentially impact on the number of MIs such as STEMI nationally.

Regarding the social determinants of health, in terms of SES, 65% of patients would be considered of below average means according to the wealth index. A similar study reported that STEMI patients from low to middle SES groups were higher among the cases and controls [13]. The paper suggested that the reasons for this could be tobacco addiction, psychosocial stress and poor food habits which are more dominant in this social class [13]. Furthermore, the majority of the patients in our study were from urban areas (74%). Incidence of CAD is associated with the urban population due to higher access to unhealthy foods and lifestyles [13].

Despite not having data regarding recreational drug use in our cohort, it is important to understand that the nature of the drug problem in Ireland is evolving quickly [26], and this may have an impact on STEMIs in young adults. A study in France found a 12.6% prevalence of recreational drug use in STEMI patients, especially those under 50 years of age [27]. Furthermore, recreational drug use was independently associated with worse in-hospital outcomes [28]. Therefore, this is another gap worth exploring given the availability of more data. The study did recommend a systematic drug test on admission of patients with STEMI to identify those with a higher risk of in-hospital cardiovascular outcomes which can be adopted in Ireland [27].

With respect to cardiovascular biomarkers, a low HDL is an indicator of developing heart disease [29]. Contributors to a low HDL include smoking, type 2 diabetes and genetic factors [30]. In this study, 78% of patients had a low HDL (< 40 mg/dL), 20% had normal levels and only 3% had the recommended HDL level of ≥ 60 mg/dL [31]. Additionally, among the patients with a low HDL, 88% had a family history of CVD. A study in Korea also showed that patients with low HDL had significantly higher in-hospital death rates than those with a normal HDL, which was partly because of pump failure in those with a low HDL [32]. The same study demonstrated that low HDL was more present in older patients, female, and those with comorbidities [32]. Also, a study in India analyzing sudden cardiac death (SCD) following a STEMI in 929 patients found that half of all the deaths were SCD, and the majority were in younger patients and occurred within the first month [33]. Although our dataset did not include mortality data, it is important to note the high incidence of follow-up procedures (64%) in our cohort and to acknowledge the risk of death from STEMI in young patients, especially those with a low HDL and a family history of CVD. In relation to LDL, a high LDL is a well-known risk factor for acute MIs, and this is similar among STEMI patients [33]. In our study, 28% had a high LDL, yet having a lower LDL is protective against atherosclerosis [33]. Although nutritional and exercise interventions have great potential in the reverse of CVD through HDL and LDL levels, not enough evidence exists to identify the most effective approaches currently [34]. Furthermore, whilst various family histories were recorded, there is also a possibility of those undetected and for example undiagnosed familial hypercholesterolaemia.

Hypertension and hyperlipidemia are the most commonly reported cardiovascular risk factors for STEMI [4]. In terms of cardiovascular risk factors, lower apoA levels are linked to a higher risk of CVD by contributing to arterial narrowing and hardening, which can lead to CAD [35].

Approximately 40% of patients in this study had diabetes classified as a HbA1c level percentage 6.5 or higher (≥ 48 mmol/mol). A study in China identified that while diabetes was a significant risk factor for CHD, the incidence in younger STEMI patients was not significantly higher than older patients. Nevertheless, other studies have reported on non-diabetic acute myocardial infarction (AMI) patients having higher blood glucose levels, low glucose intolerance or insulin resistance [16]. Additionally, diabetes is a strong predictor for cardiac outcomes, particularly for women [3].

## Limitations

This study is a single-centre, retrospective case series report, using secondary data of patients presenting with STEMI over an 8-year period and therefore is subject to the standard limitations of any retrospective, observational study [24]. The major limitations of this study are small sample size, single-group design, missing data and missing variables beyond the hospital index [13]. Due to the low incidence of STEMIs in young adults, the study sample size was quite small, despite the 8-year period for which the data was collected, and therefore the findings are not fully generalizable. However, these results are significantly applicable and important to the Mid-West community in raising awareness of STEMI in young patients. The single-group design threat [36] may result in unidentified confounders, limits the statistical tests that can be performed, and reduces the ability for cause-effect [37] relationships to be determined [38]. As the data was originally collected to analyze PCI being performed on the young STEMI patients, not all variables with potential to impact the outcome were collected such as lifestyle behaviours and complete mortality data. Additionally, variation in missing data across variables may have reduced the precision of our estimates. There was also a possibility of recall bias as patients completing the questionnaires had to recall family history details. This study could not support patients being engaged in designing, conducting, analyzing, reporting, or disseminating any of the findings in this research [24]. However, Public Patient Involvement (PPI) may be more critical during the formation of interventions for this cohort, as being a small cohort, their contribution would be fundamental for research integrity [39]. Furthermore, in Ireland, in 2020, the number of deaths to MIs was equal across males and females [6] yet the data set comprises of 90% males and only 10% females. Notwithstanding, this study may serve as a clinical and public health reference for key stakeholders to understand the risk factors in young STEMI patients and henceforth develop early preventative interventions [16].

## Conclusions

Internationally, STEMI in young adults is a growing health, social and economic problem. This study was important to explore risk factors of young patients presenting with STEMI in Ireland, in order to suggest evidence-based public health measures that could be used upstream contemporaneously with medical treatment to prevent premature deaths, excess burden on the healthcare system and productivity loss. This study determined the prevalence of cardiovascular risk factors among young STEMI patients aged 45 and below which included male sex, smoking, overweight/obesity, family history of CVD, low HDL levels and high LDL levels. Of note in this study was the fact that even patients who did not have a family history of CVD but were smokers had a STEMI which emphasizes the key role of lifestyle factors in cardiovascular health and the importance of health promotion strategies. The findings of this study will complement prospective research on STEMI in Ireland perhaps with a larger cohort in order to derive more significant statistical evidence. This study will also support the current reform to prevent avoidable CVD and reduce its burden on the Irish healthcare system.

## Electronic supplementary material

Below is the link to the electronic supplementary material.

Supplementary file1 (DOCX 24 KB)

## Data Availability

All data generated or analyzed during this study are included in this published article and its supplementary files.

## Appendix

STROBE Statement—checklist of items that should be included in reports of observational studies* to be updated last.

**Table:**

		Item No	Recommendation	Page No
Title and abstract		1	(*a*) Indicate the study’s design with a commonly used term in the title or the abstract	1,2
			(*b*) Provide in the abstract an informative and balanced summary of what was done and what was found	2
Introduction				
Background/rationale		2	Explain the scientific background and rationale for the investigation being reported	3
Objectives		3	State specific objectives, including any prespecified hypotheses	3
Methods				
Study design		4	Present key elements of study design early in the paper	4
Setting		5	Describe the setting, locations, and relevant dates, including periods of recruitment, exposure, follow-up, and data collection	4,5
Participants		6	(*a*) *Cohort study*—Give the eligibility criteria, and the sources and methods of selection of participants. Describe methods of follow-up *Case–control study*—Give the eligibility criteria, and the sources and methods of case ascertainment and control selection. Give the rationale for the choice of cases and controls *Cross-sectional study*—Give the eligibility criteria, and the sources and methods of selection of participants	4
			(*b*) *Cohort study*—For matched studies, give matching criteria and number of exposed and unexposed *Case–control study*—For matched studies, give matching criteria and the number of controls per case	N/A
Variables		7	Clearly define all outcomes, exposures, predictors, potential confounders, and effect modifiers. Give diagnostic criteria, if applicable	4
Data sources/measurement		8*	For each variable of interest, give sources of data and details of methods of assessment (measurement). Describe comparability of assessment methods if there is more than one group	5
Bias		9	Describe any efforts to address potential sources of bias	11
Study size		10	Explain how the study size was arrived at	4
Quantitative variables		11	Explain how quantitative variables were handled in the analyses. If applicable, describe which groupings were chosen and why	4,5
Statistical methods		12	(*a*) Describe all statistical methods, including those used to control for confounding	4
			(*b*) Describe any methods used to examine subgroups and interactions	4
			(*c*) Explain how missing data were addressed	5
			(*d*) *Cohort study*—If applicable, explain how loss to follow-up was addressed *Case–control study*—If applicable, explain how matching of cases and controls was addressed *Cross-sectional study*—If applicable, describe analytical methods taking account of sampling strategy	N/A
			(*e*) Describe any sensitivity analyses	N/A
Results				
Participants	13*	(a) Report numbers of individuals at each stage of study—e.g. numbers potentially eligible, examined for eligibility, confirmed eligible, included in the study, completing follow-up, and analysed		N/A
		(b) Give reasons for non-participation at each stage		N/A
		(c) Consider use of a flow diagram		N/A
Descriptive data	14*	(a) Give characteristics of study participants (e.g. demographic, clinical, social) and information on exposures and potential confounders		5–7
		(b) Indicate number of participants with missing data for each variable of interest		5
		(c) *Cohort study*—Summarise follow-up time (e.g., average and total amount)		N/A
Outcome data	15*	*Cohort study*—Report numbers of outcome events or summary measures over time		N/A
		*Case–control study—*Report numbers in each exposure category, or summary measures of exposure		5–7
		*Cross-sectional study—*Report numbers of outcome events or summary measures		N/A
Main results	16	(*a*) Give unadjusted estimates and, if applicable, confounder-adjusted estimates and their precision (e.g., 95% confidence interval). Make clear which confounders were adjusted for and why they were included		5–9
		(*b*) Report category boundaries when continuous variables were categorized		5–9
		(*c*) If relevant, consider translating estimates of relative risk into absolute risk for a meaningful time period		N/A
Other analyses	17	Report other analyses done—e.g. analyses of subgroups and interactions, and sensitivity analyses		N/A
Discussion				
Key results	18	Summarise key results with reference to study objectives		9–11
Limitations	19	Discuss limitations of the study, taking into account sources of potential bias or imprecision. Discuss both direction and magnitude of any potential bias		11–12
Interpretation	20	Give a cautious overall interpretation of results considering objectives, limitations, multiplicity of analyses, results from similar studies, and other relevant evidence		9–11
Generalisability	21	Discuss the generalisability (external validity) of the study results		11
Other information				
Funding	22	Give the source of funding and the role of the funders for the present study and, if applicable, for the original study on which the present article is based		N/A

*Give information separately for cases and controls in case–control studies and, if applicable, for exposed and unexposed groups in cohort and cross-sectional studies

Note: An Explanation and Elaboration article discusses each checklist item and gives methodological background and published examples of transparent reporting. The STROBE checklist is best used in conjunction with this article (freely available on the Web sites of PLoS Medicine at http://www.plosmedicine.org/, Annals of Internal Medicine at http://www.annals.org/, and Epidemiology at http://www.epidem.com/). Information on the STROBE Initiative is available at www.strobe-statement.org

## Abbreviations

*AMI*: Acute myocardial infarction
*apoA*: Apolipoprotein A
*CAD*: Coronary artery disease
*CVD*: Cardiovascular disease
*CHD*: Coronary heart disease
*DALY*: Disability adjusted life years
*ESC*: European Society of Cardiology
*FH*: Family history
*HbA1c*: Glycated hemoglobin
*HDL*: High density lipoproteins
*IHAA*: Irish Heart Attack Audit
*IQR*: Interquartile range
*LDL*: Low density lipoproteins
*MI*: Myocardial infraction
*mg/dL*: Milligrams per deciliter
*NCD*: Non-communicable diseases
*NSTEMI*: Non-ST-elevation myocardial infarctions
*PCI*: Percutaneous coronary intervention
*PPI*: Public Patient Involvement
*SCD*: Sudden cardiac death
*SD*: Standard deviation
*SES*: Socioeconomic status
*STEMI*: ST-elevation myocardial infarctions
*TC*: Total cholesterol
*TG*: Thyroglobulin
*TILDA*: The Irish Longitudinal Study on Ageing
*UL*: University of Limerick
*UHL*: University Hospital Limerick
