# Supplementary material for: Risk factors associated with ST-segment elevation myocardial infarctions among young patients treated in the Mid-West of Ireland: a case series report using secondary data
Source: Ir J Med Sci. 2025 May 29;194(4):1267–77. doi: 10.1007/s11845-025-03965-w (PMC12413419; doi:10.1007/s11845-025-03965-w)
Supplement: Supplementary file 1 — Supplementary file1 (DOCX 24 KB) [file 11845_2025_3965_MOESM1_ESM.docx]

# **Supplementary Tables**

Supplementary Table 1: Characteristic Comparison Across Family History of CVD

|  | | **No Family History CVD** | | **Family History CVD** | | **p-Value** |
| --- | --- | --- | --- | --- | --- | --- |
| **Characteristic** | **Detail** | **n** | **%** | **n** | **%** |  |
| **Sex** (n=78) | Female | 2 | 11% | 5 | 11% | 0.899 |
|  | Male | 17 | 89% | 38 | 88% |  |
| **Age (y)** (n=78) | 31-35 | 0 | 0% | 4 | 9% | 0.385 |
|  | 36-40 | 6 | 32% | 13 | 30% |  |
|  | 41-45 | 13 | 68% | 26 | 61% |  |
| **BMI** (n=101) | Healthy | 7 | 37% | 15 | 34% | 0.882 |
|  | Overweight or Obese | 12 | 63% | 28 | 65% |  |
| **Smoking Status** (n=39) | Non- smoker | 3 | 23% | 8 | 31% | 0.715 |
|  | Smoker | 9 | 69% | 18 | 69% |  |
| **Residence** (n=39) | Rural | 5 | 39% | 5 | 19% | 0.195 |
|  | Urban | 8 | 62% | 21 | 81% |  |
| **SES** (n=39) | Above Average | 4 | 31% | 10 | 39% | 0.769 |
|  | Below Average | 9 | 69% | 16 | 63% |  |
| **Readmission** (n=78) | Yes | 15 | 79% | 25 | 58% | 0.114 |
|  | No | 4 | 21% | 18 | 42% |  |
| **Hypertension** (n=66) | No | 10 | 59% | 21 | 49% | 0.485 |
|  | Yes | 7 | 41% | 22 | 51% |  |
| **FHxCAD** (n=62) | Yes | 1 | 5% | 23 | 54% | <0.05* |
|  | No | 18 | 95% | 20 | 47% |  |
| **FHx Hypercholesterolaemia** (n=60) | Yes | 2 | 11% | 20 | 49% | <0.05* |
|  | No | 17 | 90% | 21 | 51% |  |
| **HbA1c (%)** (n=71) | Normal (<5.7) | 0 | 0% | 15 | 36% | 0.019* |
|  | Pe-diabetes (5.7-6.4) | 9 | 60% | 13 | 40% |  |
|  | Diabetes (≥6.5) | 6 | 40% | 27 | 33% |  |
| **ApoA (mg/dl)** (n=78) | High Risk (<110) | 13 | 68% | 19 | 63% | 0.101 |
|  | Normal  (110-180) | 6 | 32% | 8 | 18.6% |  |
|  | Recommended (>180) | 0 | 0% | 8 | 18.6% |  |
| **HDL (mg/dl)** (n=78) | High Risk (<40) | 12 | 63% | 38 | 88% | <0.05** |
|  | Normal (40-59) | 6 | 32% | 5 | 12% |  |
|  | Recommended  (≥60) | 1 | 5% | 0 | 0% |  |
| **LDL (mg/dl)** (n=78) | Optimal (<100) | 10 | 56% | 22 | 51% | 0.055 |
|  | Near Optimal (100-129) | 3 | 17% | 9 | 21% |  |
|  | Borderline High (130-159) | 2 | 11% | 9 | 21% |  |
|  | High (160-189) | 0 | 0% | 3 | 7% |  |
|  | Very High(≥190) | 3 | 17% | 0 | 0% |  |

Supplemetary Table 2: Characteristic Comparison Across Diabetes Status

|  | | **No Diabetes** | | **Pre-Diabetes** | | **Diabetes** | | **p-Value** |
| --- | --- | --- | --- | --- | --- | --- | --- | --- |
| **Characteristic** | **Detail** | **n** | **%** | **n** | **%** | **n** | **%** |  |
| **Sex** (n=78) | Female | 2 | 11% | 1 | 4% | 2 | 7% | 0.720 |
|  | Male | 17 | 89% | 23 | 96% | 26 | 93% |  |
| **Age (y)** (n=78) | 31-35 | 2 | 11% | 1 | 4% | 0 | 0% | 0.136 |
|  | 36-40 | 6 | 32% | 4 | 17% | 12 | 43% |  |
|  | 41-45 | 11 | 56% | 19 | 79% | 16 | 57% |  |
| **BMI** (n=101) | Healthy | 8 | 42% | 9 | 38% | 6 | 21% | 0.267 |
|  | Overweight or Obese | 11 | 58% | 15 | 63% | 22 | 79% |  |
| **Smoking Status** (n=39) | Non- smoker | 4 | 57.1% | 3 | 23.1% | 4 | 22.2% | 0.190 |
|  | Smoker | 3 | 42.9% | 10 | 76.9% | 14 | 77.8% |  |
| **Residence** (n=39) | Rural | 2 | 28.6% | 4 | 28.6% | 4 | 22.2% | 0.903 |
|  | Urban | 5 | 71.4% | 10 | 71.4% | 14 | 77.8% |  |
| **SES** (n=39) | Above Average | 3 | 42.9% | 5 | 35.7% | 6 | 33.3% | 0.898 |
|  | Below Average | 4 | 57.1% | 9 | 64.3% | 12 | 66.7% |  |
| **Readmission** (n=78) | Yes | 13 | 68% | 16 | 67% | 14 | 50% | 0.337 |
|  | No | 6 | 32% | 8 | 33% | 14 | 50% |  |
| **Hypertension** (n=66) | No | 9 | 50% | 8 | 35% | 12 | 50% | 0.498 |
|  | Yes | 9 | 50% | 15 | 65% | 12 | 50% |  |
| **FHxCAD** (n=62) | No | 7 | 47% | 12 | 55% | 15 | 75% | 0.197 |
|  | Yes | 8 | 53% | 10 | 46% | 5 | 25% |  |
| **FHx Hypercholesterolaemia** (n=60) | Yes | 7 | 50% | 9 | 41% | 6 | 32% | 0.562 |
|  | No | 7 | 50% | 13 | 59% | 13 | 68% |  |
| **ApoA (mg/dl)** (n=78) | High Risk (<110) | 12 | 68% | 14 | 58% | 26 | 93% | 0.021* |
|  | Normal  (110-180) | 3 | 32% | 7 | 29% | 2 | 7% |  |
|  | Recommended (>180) | 4 | 0% | 3 | 13% | 0 | 0% |  |
| **HDL (mg/dl)** (n=78) | High Risk (<40) | 17 | 90% | 16 | 70% | 19 | 76% | 0.180 |
|  | Normal (40-59) | 2 | 11% | 7 | 30% | 4 | 16% |  |
|  | Recommended  (≥60) | 0 | 0% | 0 | 0% | 2 | 8% |  |
| **LDL (mg/dl)** (n=78) | Optimal (<100) | 6 | 32% | 12 | 52% | 12 | 48% | 0.080 |
|  | Near Optimal (100-129) | 8 | 42% | 4 | 17% | 5 | 20% |  |
|  | Borderline High (130-159) | 5 | 26% | 2 | 9% | 7 | 28% |  |
|  | High (160-189) | 0 | 0% | 3 | 13% | 0 | 0% |  |
|  | Very High(≥190) | 0 | 0% | 2 | 9% | 1 | 4% |  |
